# Antimeningococcal Protection in Patients Receiving Terminal Complement Inhibitors

**DOI:** 10.1016/j.ekir.2025.11.025

**Published:** 2025-11-25

**Authors:** Aleksandra Vujović, Franz Schaefer, Anne-Laure Sellier-Leclerc, Mattia Parolin, Víctor Pérez-Beltrán, Jonas Hofstetter, Olivia Boyer, Maria Cristina Mancuso, Sandra Habbig, Tanja Kersnik Levart, ERKNet TMA Working Group and Other Collaborators, Klaus Arbeiter, Fabian Eibensteiner, Lovro Lamot, Justine Bacchetta, Lisa Condamine, Dounia Habchi, Lydia Slimani, Amina Talhi, Sacha Flammier, Marc Fila, Julie Tenenbaum, Laurence Heidet, Kahina Saidoun, Nabila Moussaoui, Stefan Kohl, Susanne Schaefer, Jose Antonio Ramirez Garcia, Marcus Weitz, Kathrin Buder, Lena Lechler, Matko Marlais, Orsolya Horváth, Giovanni Montini, Francesco Trepiccione, Miriam Zacchia, Germana Longo, Nicola Bertazza Partigiani, Antonio Gargiulo, Chiara Bettini, Vitor Hugo Martins, Aleksandra Zurowska, Magdalena Drozynska-Duklas, Anna Krakowska, Joaquim Calado, Rute Baeta Baptista, Ana Teixeira, Anca Marin, Valentin Mocanu, Andreja Aleš Rigler, Tomaž Šimnovec, Carla Soto, Romy Bouwmeester, Eiske Dorresteijn

**Affiliations:** 1 Faculty of Medicine, University of Ljubljana, Ljubljana, Slovenia; 2 Division of Pediatric Nephrology, University Children’s Hospital, University Medical Centre Ljubljana, Ljubljana, Slovenia; 3 Division of Pediatric Nephrology, Department of Pediatrics, University of Heidelberg, Heidelberg, Germany; 4 Service de Néphrologie Pédiatrique, Centre de Référence Des Maladies Rénales Rares MAREGE Filières Maladies Rares ORKID et ERK-Net, Hospices Civils de Lyon, Bron, France; 5 Division of Pediatric Nephrology, Department of Women’s and Child’s Health, University Hospital of Padova, Padua, Italy; 6 Division of Pediatric Nephrology and Renal Transplantation, Hospital Universitari Vall d'Hebron, Barcelona, Spain; 7 Service de Néphrologie Pédiatrique, Dialyse et Transplantation, Hôpital, Centre de Référence MARHEA, Hôpital Necker Enfants Malades, Université Paris Cité, France; 8 Division of Pediatric Nephrology, Dialysis and Transplantation Unit, Fondazione IRCCS Ca' Granda, Ospedale Maggiore Policlinico, Milan, Italy; 9 Department of Pediatrics and Center for Family Health, University Hospital Cologne and Faculty of Medicine, University of Cologne, Cologne, Germany

**Keywords:** antibiotic prophylaxis, antimeningococcal protection, antimeningococcal vaccination, atypical hemolytic uremic syndrome, complement inhibitors, meningococcal infection

## Abstract

**Introduction:**

C5 inhibitor (C5i) therapy markedly increases susceptibility to invasive meningococcal disease (IMD) by blocking the terminal complement pathway essential for defense against *Neisseria meningitidis.* Vaccination is recommended for all recipients, yet breakthrough infections persist. Antibiotic prophylaxis is not universally endorsed, resulting in variable practices. We aimed to assess whether antibiotic prophylaxis provides additional protection beyond vaccination in C5i-treated patients.

**Methods:**

The analysis included 124 C5i recipients treated for > 6 months. Patients were classified as receiving single protection (vaccination or antibiotic prophylaxis alone) or combined protection (vaccination and continuous antibiotic prophylaxis). The outcomes were analyzed by prescribed and by implemented regimen; the latter accounting for patient adherence to antibiotic prophylaxis.

**Results:**

Of the patients, 60% were prescribed combined protection. Booster vaccination coverage was low (< 40%), and one-quarter of patients did not adhere to antibiotic prophylaxis. The overall incidence of IMD was 0.74 cases per 100 patient-years (PY) (95% confidence interval [CI]: 0.37–1.32). After accounting for noncompliance, the incidence of IMD remained significantly lower in the combined protection group (3.1 [95% CI: 1.5–4.8] vs. 0.5 [95% CI: 0.0–2.7], *P* = 0.03), corresponding to a 6-fold reduction in risk. Eleven infections were reported, predominantly due to serogroup B (45.5%). Ten patients recovered completely, and 1 had mild residual disability.

**Conclusion:**

Although guidelines recommend vaccination alone, our findings indicate that combined protection offers substantially greater protection against IMD in patients receiving long-term C5i. Continued prospective monitoring will be essential to define the optimal preventive strategies in this high-risk population.

## Graphical abstract

**Figure:**
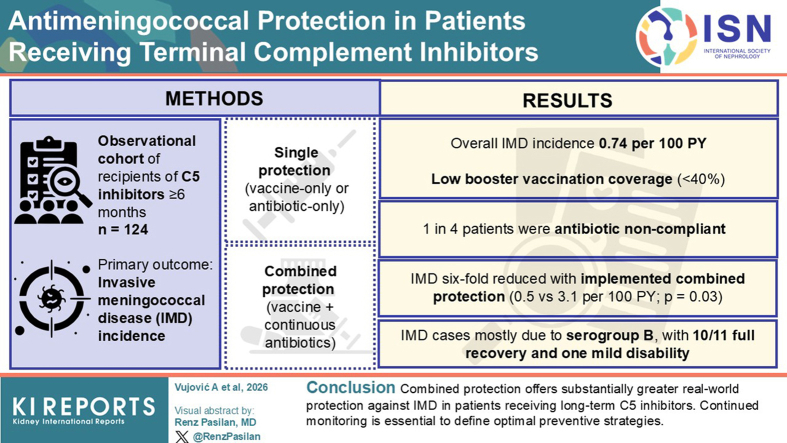

The advent of terminal complement inhibitors has markedly improved the prognosis of complement-mediated diseases. By specifically targeting the C5 component, these treatments have transformed atypical hemolytic uremic syndrome from a disease with a dismal prognosis to one with excellent patient survival, kidney function preservation, and overall quality of life.^1^^,^^2^ Although the efficacy of C5i has been demonstrated in numerous studies, several questions, in particular regarding their long-term safety, remain to be answered by extended real-world monitoring.

Eculizumab use has been associated with ≤ 2000 times increased risk of meningococcal infections.^3^ The complement system, particularly its terminal pathway, is essential in protecting against invasive *N meningitidis*. This bacterium is typically a harmless colonizer of the nasopharynx; however, under certain conditions, it may enter the bloodstream and lead to IMD. In immunocompetent individuals, the complement system plays a central role in eliminating invasive bacterial threats through the formation of the membrane attack complex. Inhibition of C5 impairs the formation of membrane attack complex, thereby diminishing the host’s capacity to eliminate *N meningitidis*.^4^

The prescribing information for both eculizumab and ravulizumab mandates vaccination against *N meningitidis* before treatment initiation to reduce the risk of IMD.^5^^,^^6^ Recombinant vaccines against serotype B (MenB) and quadrivalent vaccines against serotypes A, C, W, and Y (MenACWY) are available, with schedules tailored to age and underlying risk in immunocompetent individuals. In patients receiving C5i, who are at markedly elevated risk of IMD, booster vaccination is advised.

In 2020, the US Advisory Committee on Immunization Practices issued specific recommendations for individuals at increased risk of IMD, including C5i recipients.^7^ For those aged ≥ 10 years, MenB is advised, with a booster 1 year after the primary series, followed by boosters every 2 to 3 years if the risk persists. In contrast, the European Medicines Agency has licensed MenB vaccines for routine use from 2 months of age.^8^ For MenACWY, a 2-dose primary series is recommended, followed by 5-yearly boosters. Although the optimal timing and necessity of additional booster doses remain uncertain, patients receiving C5i are considered at elevated risk for IMD, even if postvaccination antibodies develop.^8^^,^^9^ This residual risk reflects impaired complement-mediated defense as well as disproportionate susceptibility to infections with nongroupable strains of *N meningitidis*, which are not covered by available vaccines.^3^

The prescribing information for eculizumab and ravulizumab does not recommend routine, continuous antibiotic prophylaxis in addition to vaccination. In situations where immediate initiation of C5i is required and vaccination is not feasible, temporary antibiotic prophylaxis is advised until the recommended vaccination series has been administered.^5^^,^^6^ In clinical practice, however, approaches vary; whereas some physicians rely on vaccination alone, others continue antibiotic prophylaxis parallel to vaccination.

Against this background, the present study aimed to identify the optimal antimeningococcal protection strategy in patients treated with C5i by comparing single protection (vaccination or antibiotic prophylaxis alone) with combined protection (vaccination and continued antibiotic prophylaxis). To this end, we analyzed patients treated with C5i enrolled in the European Rare Kidney Disease Registry (ERKReg), one of the largest prospective, real-world cohorts of complement mediated kidney diseases.

## Methods

This observational cohort study analyzed real-world data from ERKReg on patients receiving C5i, enrolled into the registry between January 1, 2019, and January 31, 2024. ERKReg, an initiative of the European Reference Network for Rare Kidney Diseases, compiles data from 76 specialized pediatric and adult nephrology centers across 24 countries. Data collection included demographics; diagnostic findings; concomitant treatments, such as immunosuppression, plasma exchange, and acute dialysis; as well as kidney function at the last follow-up. To complement these routinely collected registry data, participating physicians were invited to complete an additional patient-level survey for the same registered patients between May 17, 2023, and January 31, 2024, providing detailed information on antimeningococcal protection strategies, adherence, and infection outcomes.

**Figure 1:**
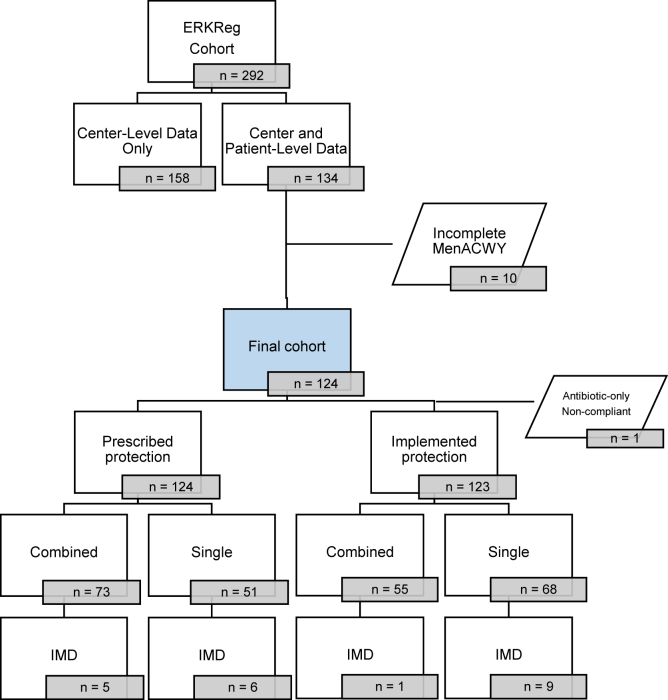
Flow chart showing the selection of patients from ERKReg for the analytic cohort. Of 292 patients receiving C5 inhibitors for > 6 months, 134 had detailed patient-level data. After excluding ten patients without MenACWY vaccination, 124 patients remained. These were divided into prescribed combined (vaccination and continued antibiotic prophylaxis; *n* = 73) and single protection (vaccination or antibiotic prophylaxis only, *n* = 51). The implemented protection analysis included 123 patients after excluding one non-compliant antibiotic-only case, yielding implemented combined (n = 55) and single (n = 68) protection groups. 11 IMD cases occurred in total. ERKReg, European Rare Kidney Disease Registry; IMD, invasive meningococcal disease; MenACWY, quadrivalent meningococcal conjugate vaccine; MenB, meningococcal serogroup B vaccine.

Patients with ≥ 6 months of C5i exposure were eligible. In total, 292 patients were identified, as shown in Figure 1. For 158 patients, centers provided only information on the presence or absence of IMD, whereas 134 patients with completed survey data were included in the main analysis. Out of these 134 patients, 10 were excluded, because they had not received MenACWY (7 received MenB with antibiotic prophylaxis and 3 MenB only). This yielded a final cohort of 124 patients, categorized into single protection (vaccination or antibiotic prophylaxis only) and combined protection group (vaccination and continued antibiotic prophylaxis). Patients receiving antibiotic prophylaxis only until completion of the vaccination series were classified under single protection.

The analysis was performed in 2 parts. First, we evaluated the prescribed antimeningococcal protection strategy. Second, we analyzed the implemented protection, accounting for compliance with antibiotic prophylaxis as reported by treating physicians. Antibiotic compliance was graded on the following 5-stage scale: 1 = noncompliant, 2 = barely compliant, 3 = partially compliant, 4 = well-compliant, and 5 = fully compliant. For analytical purposes, levels 1 to 3 were categorized as noncompliant and levels 4 and 5 as compliant. In the second part of the analysis, patients rated noncompliant were reassigned from the combined to the single protection group. One patient with IMD in the single protection group, who had received antibiotic prophylaxis only but was noncompliant, was excluded. This yielded a final implemented cohort of 123 patients.

The primary outcome was the occurrence of IMD, as reported by participating centers and defined according to local clinical and microbiological criteria. The main exposure was the antimeningococcal protection strategy at the time of infection or last follow-up, categorized as either single or combined protection. Additional predictors and risk factors included age, sex, underlying diagnosis (e.g., atypical hemolytic uremic syndrome, C3 glomerulopathy), use of concomitant immunosuppressive therapy, need for acute dialysis at initial disease onset, and kidney function at last follow-up. Compliance with antibiotic prophylaxis was considered a potential effect modifier, enabling distinction between prescribed and implemented protection strategies. Comparisons between protection strategies were based on incidence rates per 100 PY of C5i exposure, thereby accounting for differences in treatment duration and follow-up across groups.

Chronic kidney disease (CKD) stage was determined from available glomerular filtration rate data, calculated using the Schwartz formula as implemented in the ERKReg. Blood pressure measurements were available in some cases; however, information on proteinuria, hematuria, or other markers of kidney damage was not consistently captured. Acute kidney injury was classified according to Kidney Disease: Improving Global Outcomes criteria; patients requiring dialysis were categorized as having acute kidney injury requiring dialysis. Staging according to Kidney Disease: Improving Global Outcomes was limited by the absence of urine output data and was therefore based on glomerular filtration rate and the need for renal replacement therapy. All infections were confirmed by positive blood cultures with strain identification, except in a single case diagnosed by polymerase chain reaction alone. Information on the clinical presentation of infection, including manifestations such as meningitis or sepsis, was incomplete and therefore not systematically analyzed.

The reporting of this study follows the principles outlined in the Strengthening the Reporting of Observational Studies in Epidemiology guidelines. In line with sex- and gender-based analysis recommendations, sex was recorded as a binary variable (male/female) based on investigator-reported data corresponding to sex assigned at birth. Gender identity was not collected.

### Statistics

Continuous variables were summarized as means with SD or as medians with interquartile ranges, according to their distribution. Categorical variables were reported as absolute counts (*n*) and percentages. Associations between categorical variables were assessed using the chi-square test, with Fisher exact test applied when expected cell counts were < 5. A *P*-value ≤ 0.05 was considered statistically significant. Statistical significance was defined as a 2-sided *P*-value ≤ 0.05. Infection-free survival was estimated by Kaplan–Meier analysis. Because the number of meningococcal infection events was low, infection-free survival was analyzed in all models using Cox regression with Firth’s penalized likelihood correction to reduce small-sample bias and ensure finite coefficient estimates. Missing data were not imputed; all analyses were conducted on complete-case data.

### Ethics Approval

Each participating center contributing data to ERKReg obtained approval from its local ethics committee. Written informed consent for inclusion in the registry was obtained from patients or their legal guardians, as required. This study was conducted in accordance with the ethical standards of the institutional and national research committees, as well as the 1964 Declaration of Helsinki and its later amendments. Ethical approval for this analysis was granted by the Republic of Slovenia National Medical Ethics Committee (Komisija Republike Slovenije za medicinsko etiko) under approval number 0120-224/2023/3, issued on 11 July 2023.

## Results

### Cohort Description

A total of 124 patients treated with C5i for > 6 months were included, contributed by 26 specialized centers across 13 European countries. At the time of the analysis, 117 patients were receiving eculizumab and 7 ravulizumab. Baseline characteristics are summarized in Table 1. The 2 groups were comparable regarding sex, age, need for dialysis at disease onset, underlying diagnoses (atypical hemolytic uremic syndrome, C3 glomerulopathy, secondary hemolytic uremic syndrome), concomitant immunosuppression, and follow-up duration. Dual protection was prescribed more frequently in pediatric patients (*P* = 0.05) and in those with early-stage CKD (*P* = 0.03).

**Table 1:** Patient characteristics by prescribed antimeningococcal protection: Combined vs single

Characteristics	Combined (both vaccination and antibiotic prophylaxis)	Single (either vaccination or antibiotic prophylaxis)	*P-* value
*n*	73/124 (58.9 %)	51/124 (41.1%)	
Female, *n*	33/72 (45.2 %)	21/51 (41.2%)	0.74
Age at initial disease onset (yr)	7.7 (0.1-56.8)	10.6 (0.04-45.0)	0.16
Adult-onset of initial disease	2/56 (3.6%)	8/51 (15.7%)	0.05^a^
Follow-up (yr)	6.8 (0.5-20.5)	8.3 (0.5-30.9)	0.20
AKI-D at initial disease onset, *n* (%)	36/69 (52.2 %)	27/50 (54.0%)	0.99
aHUS	60/73 (82.2%)	45/51 (88.2%)	0.51
Immunosuppressive therapy (other than C5i)	19/60 (31.7%)	15/45 (33.3%)	1.00
Secondary HUS	4/73 (5.5%)	1/51 (2.0%)	0.65
Immunosuppressive therapy (other than C5i)	2/4 (50.0%)	0/1 (0%)	1.00
C3 glomerulopathy	9/73 (12.3%)	5/51 (9.8%)	0.88
Immunosuppressive therapy (other than C5i)	8/9 (88.9%)	2/5 (40.0%)	0.09
% CKD 1 – 2 – 3 – 4 – 5 - 5D/Tx at last follow-up	57.1 / 10.0 / 7.1 / 1.4 / 0 / 11.4	43.5 / 28.3 / 8.7 / 4.3 / 2.2 / 13.0	0.03^a^

aHUS, atypical HUS; AKI-D, acute kidney injury requiring dialysis; C3G, C3 glomerulopathy; C5i, C5 inhibitor; CKD, chronic kidney disease; HUS, hemolytic uremic syndrome.

Data availability for combined protection group: sex 72/73; age at initial disease onset 56/73; adult-onset of initial disease 56/73; follow-up 62/73; AKI-D at initial disease onset 69/73, kidney outcome at last follow-up 70/73; diagnosis (aHUS, C3G, secondary HUS) 73/73; immunosuppressive therapy (other than C5i) 73/73.

Data availability for single protection group: sex 51/51; age at initial disease onset 46/51; adult-onset of initial disease 46/51; follow-up 48/51; AKI-D at initial disease onset 50/51, kidney outcome at last follow-up 46/51; diagnosis (aHUS, C3G, secondary HUS) 51/51; immunosuppressive therapy (other than C5i) 51/51.

Data are given as *n* (%) or median (range) as appropriate.

Immunosuppressive therapy included administration of glucocorticoids, calcineurin inhibitors, antimetabolites, and biological agents.

*P*-values indicate between-group comparisons; statistically significant values (*P* < 0.05) are marked with superscript letters.

### Antimeningococcal Protection

The use of antimeningococcal vaccines and antibiotic prophylaxis based on prescribed regimens is summarized in Table 2. Of 124 patients, 73 (58.9%) were prescribed combined protection and 51 (41.1%) single protection. The number of administered MenB and MenACWY primary and booster doses was similar across groups. Likewise, the proportion of patients who were nonadherent to antibiotic prophylaxis did not differ substantially between the groups. Of 124 patients, 84 were prescribed antibiotic prophylaxis, of these, 19 were categorized as noncompliant (5 noncompliant, 3 barely compliant, and 11 partially compliant) and 65 as compliant (23 well-compliant and 42 fully compliant). Duration of antibiotic prophylaxis was significantly longer in the combined protection group. After accounting for adherence and excluding 1 noncompliant patient from the single protection group (antibiotic prophylaxis only), the implemented regimens included 55 patients (44.7%) in the combined protection group and 68 (55.3%) in the single protection group. Implemented vaccination protocols, as summarized in Table 3, were comparable between groups.

**Table 2:** Utilization of vaccines and antibiotics according to prescription of combined or single antimeningococcal protection

	Combined (both antibiotics and vaccination)	Single (either vaccination or antibiotics)		*P*-value
		Vaccination (40)	Antibiotic (11)	
*n*	73/124 (58.9%)	51/124 (41.1%)		
MenB immunization: *n* of primary vaccinations	73/73 (100%)	40/40 (100%)		
*n* = 2	47/73 (64.4%)	31/40 (77.5.8%)		0.29
*n* = 3	16/73 (21.9%)	6/40 (15.0%)		0.52
Uncertain *n* of doses	10/73 (13.7%)	3/40 (7.5%)		0.38
MenB booster doses *n/n*^0^	3/25 (12.0%)	3/17 (17.6%)		0.67
MenACWY immunization: *n* of primary vaccinations	73/73 (100%)	40/40 (100%)		
*n* = 1	51/73 (69.9%)	32/40 (80.0%)		0.34
*n* = 2	16/73 (21.9%)	6/40 (15.0%)		0.52
Uncertain *n* of doses	6/73 (8.2%)	2/40 (5.0%)		0.71
MenACWY booster doses, *n/n*^0^	9/22 (40.9%)	5/18 (27.8%)		0.65
Median (range) duration of antibiotic use (yr)	4.7 (0.3–15.0)		2.9 (0.3–11.2)	< 0.001
Noncompliance with antibiotic use	18/73 (24.7%)		1/11 (9.0%)	0.44

MenACWY, vaccines against serotypes A, C, Y, and W; MenB, vaccines against serotype B; *n*, counts; *n*^0^, *n* of patients requiring booster doses according to the vaccination regime; primary vaccination series, all initial vaccination doses before the booster dose.

Data availability for combined protection group: MenB primary vaccination series 63/73; MenB booster doses 25/30; MenACWY primary vaccination series 67/73; MenACWY booster doses 22/26; Duration of antibiotic use 67/73; Non-compliance to antibiotic use 73/73.

Data availability for single protection group: Men B primary vaccination series 37/40; MenB booster doses 17/20; MenACWY primary vaccination series 38/40; MenACWY booster doses 18/20; Duration of antibiotic use 11/11; Non-compliance to antibiotic 11/11.

**Table 3:** Utilization of vaccines and antibiotics according to implementation of combined or single antimeningococcal protection

	Combined (both antibiotics and vaccination)	Single (either vaccination or antibiotics)		*P*-value
		Vaccination (58)	Antibiotic (10)	
*n*	55/123 (44.7%)	68/123 (55.3%)		
MenB immunization: *n* of primary vaccinations	55/55 (100%)	58/58 (100%)		
*n* = 2	33/55 (60.0%)	45/58 (77.6%)		0.07
*n* = 3	15/55 (27.3%)	7/58 (12.1%)		0.07
Uncertain *n* of doses	7/55 (12.7%)	6/58 (10.3%)		0.92
MenB booster doses *n/n*^0^	2/21 (9.5%)	4/21 (19.0%)		0.67
MenACWY immunization: *n* of primary vaccinations	55/55 (100%)	58/58 (100%)		
*n* = 1	37/55 (67.3%)	46/58 (79.3%)		0.21
*n* = 2	14/55 (25.4%)	8/58 (3.8%)		0.18
Uncertain *n* of doses	4/55 (7.3%)	4/58 (6.9%)		1.00
MenACWY booster doses, *n/n*^0^	6/18 (33.3%)	7/22 (31.8%)		0.97
Median (range) duration of antibiotic use (yr)	4.3 (0.3–12.8)		2.9 (0.3–11.2)	0.10

MenACWY, vaccines against serotypes A, C, Y, and W; MenB -, vaccines against serotype B; *n*, counts; *n*^0^, *n* of patients requiring booster doses according to the vaccination regime; primary vaccination series, all initial vaccination doses before the booster dose.

Data availability for combined protection group: MenB primary vaccination series 48/55; MenB booster doses 21/24; MenACWY primary vaccination series 51/55; MenACWY booster doses 18/20; Duration of antibiotic use 51/55.

Data availability for single protection group: Men B primary vaccination series 52/58; MenB booster doses 21/26; MenACWY primary vaccination series 54/58; MenACWY booster doses 22/26; Duration of antibiotic use 10/10.

### Rate of Infection

In the full cohort of 292 patients treated with C5i for > 6 months, the cumulative C5i exposure amounted to 1494.30 PY. In total, 11 of the 292 patients experienced IMD, corresponding to an overall incidence of 3.76% or 0.74 IMD cases per 100 PY (95% CI: 0.37–1.32).

For the analytic cohort of 124 patients with complete follow-up data, incidence rates by prescribed protection are shown in Table 4. The number of IMD cases was comparable across prescribed protection, underlying diagnoses, concomitant immunosuppression, and cumulative C5i exposure. Accordingly, the incidence rate of IMD per 100 PY on C5i did not differ significantly between the prescribed combined and single protection groups (*P* = 0.25).

**Table 4:** Incidence rate of invasive meningococcal disease (IMD) in prescribed combined and single antimeningococcal protection groups

	Combined (both antibiotics and vaccination)	Single (either antibiotics or vaccination)	*P*-value
*n*	73/124 (58.9%)	51/124 (41.1%)	
*n* of IMD cases	5/73 (6.8%)	6/51 (11.8%)	0.53
*n* of IMD per diagnosis (aHUS; C3G; secHUS)	4; 1; 0	5; 1; 0	1.00
*n* of IMD with immunosuppression (other than C5i)	2/5 (40.0%)	6/6 (100%)	0.06
Median (Q1–Q3) C5i duration per patient in yrs	2.6 (1.1–6.0)	3.0 (1.0–6.1)	0.54
IMD incidence rate per 100 PY on C5i (95% CI)	1.8 (0.2–3.4)	2.7 (0.5–4.9)	0.25

aHUS, atypical hemolytic uremic syndrome; C3G, C3 glomerulopathy; C5i, C5 inhibitor; CI, confidence interval; IMD, invasive meningococcal disease; *n*, counts; PY, patient-years; secHUS, secondary hemolytic uremic syndrome.

Immunosuppression included administration of corticosteroids, calcineurin inhibitors, antimetabolites, and biological agents.

One patient with IMD was excluded from the implemented analysis because of noncompliance with the prescribed single (antibiotic) regimen. When stratified by implemented protection, only 1 IMD occurred among patients under combined protection, compared with 9 cases under single protection (*P* = 0.044). This translated into a statistically significant 6-fold lower incidence rate (0.5 vs. 3.1 cases per 100 PY [*P* = 0.03]) in the combined protection group, as shown in Table 5.

**Table 5:** Incidence rate of invasive meningococcal disease (IMD) in implemented combined and single antimeningococcal protection groups

	Combined (both antibiotics and vaccination)	Single (either antibiotics or vaccination)	*P*-value
*n*	55/123 (44.7%)	68/123 (55.3%)	
*n* of IMD cases	1/55 (1.8%)	9/68 (13.2%)	0.044
*n* of IMD cases per diagnosis (aHUS; C3G; secHUS)	1; 0; 0	7; 2; 0	1.00
*n* of IMD with immunosuppression (other than C5i)	0/1 (0%)	7/9 (77.8%)	0.30
Median (Q1–Q3) C5i duration per patient in yrs	2.6 (1.0–6.0)	3.0 (1.0–5.8)	0.55
IMD Incidence rate per 100 PY on C5i (95% CI)	0.5 (0.0–2.7)	3.1 (1.5–4.8)	0.03

aHUS, atypical hemolytic uremic syndrome; C3G, C3 glomerulopathy; C5i, C5 inhibitor; CI, confidence interval; IMD, invasive meningococcal disease; *n*, counts; PY, patient-years; secHUS, secondary hemolytic uremic syndrome.

Immunosuppression included administration of corticosteroids, calcineurin inhibitors, antimetabolites, and biological agents.

**Figure 2:**
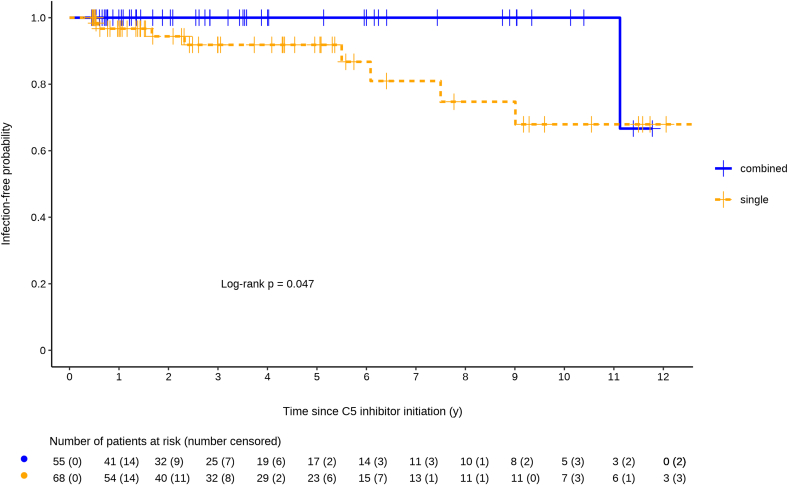
Infection-free survival following C5 inhibitor initiation in implemented combined and single antimeningococcal protection groups. This figure shows the probability of remaining infection-free over time, starting from C5-inhibitor initiation, for 2 groups based on antimeningococcal protection: combined protection (blue) and single protection (orange).

The Kaplan-Meier analysis, presented in Figure 2, depicts the infection-free survival probability in patients receiving either combined or single protection. Although both groups exhibited high early infection-free survival probabilities, the curve for single protection declined more steeply over time, reflecting a higher cumulative burden of IMD. In contrast, the combined protection group maintained a more sustained infection-free survival probability, suggesting a protective advantage (log-rank *P* = 0.047).

In the univariable model, single protection was associated with a higher risk of infection compared to combined protection (hazard ratio = 4.47, 95% CI: 1.003–42.020; Figure 2). This association was no longer evident after adjustment for either concomitant immunosuppression (hazard ratio = 3.78, 95% CI: 0.84–35.78) or for primary diagnosis (hazard ratio = 3.96, 95% CI: 0.86–37.80) in 2 separate multivariable models.

### IMD Cases

Eleven patients (9 with atypical hemolytic uremic syndrome and 2 with C3 glomerulopathy) in our cohort developed IMD. The median (range) age at onset of the underlying disease was 12.3 (2.4–19.6) years, and the median (range) age at the time of IMD was 17.7 (11.4–20.2) years.

As shown in Figure 1, 6 out of 11 patients with IMD had been prescribed single protection: 3 through vaccination alone and 3 through antibiotic prophylaxis alone, with poor adherence reported in 1 case. The remaining 5 patients had been prescribed combined protection, of whom 4 were noncompliant with antibiotic prophylaxis. Overall, only 1 patient with IMD had implemented combined protection, 9 had implemented single protection, and 1 had received no effective protection at all. The latter case was excluded from further analysis.

All 8 vaccinated patients with IMD (3 with single and 5 with combined prescribed protection; 7 with single and 1 with combined implemented protection) received standard vaccination with both MenB and MenACWY vaccines. Of these, 6 patients had received booster doses: 4 with a MenACWY booster only, and 2 with both MenB and MenACWY boosters. The timing of the booster was unknown for 1 patient, whereas in the remaining 5, boosters were administered a median (range) of 5.6 (4.1–8.3) years after the primary vaccination. All patients with IMD who were prescribed antibiotic prophylaxis (3 with single and 5 with combined prescribed protection; 7 with single and 1 with combined implemented protection) received penicillin-class antibiotics, whereas the exact dosage were not known.

Breakthrough infections in the implemented protection groups occurred at the median (range) of 5.5 (0.5–13.3) years from C5i initiation. Among 9 patients with implemented single protection, infections occurred at a median (range) of 3.9 (0.5–13.3) years, whereas in the single case with implemented combined protection, infection developed 11.1 years after C5i initiation.

At the time of infection, patients’ CKD stage ranged from 1 to 2. Concomitant immunosuppression among 8 implemented single protection patients included corticosteroids alone in 3 patients, calcineurin inhibitors in 2, antimetabolites combined with corticosteroids in 1, and triple therapy (corticosteroids, antimetabolites, and calcineurin inhibitors) in 2. The single patient with implemented combined protection did not receive any additional immunosuppression. Notably, among 11 kidney transplant recipients in our cohort, only 1 experienced IMD, occurring under implemented single protection.

All 11 patients with IMD presented with fever. Headache was reported in 10, vomiting and nausea in 8, stiff neck in 6, and photophobia in 5. Altered mental status and petechial rash were each reported in 4 patients. Severe muscle or joint pain and cold extremities were noted in 2 patients each. Additional symptoms, including abdominal pain, diarrhea, pallor, tachypnea, and disseminated intravascular coagulation, were each reported in 1 case.

Serogroup identification revealed 5 infections because of serogroup B, 2 because of serogroup Y, and 1 each because of serogroups C, W, and X. In 1 case, the serogroup remained unidentified. Antimicrobial resistance was documented in 3 patients, all of whom were receiving penicillin prophylaxis as part of prescribed combined protection; 2 of these were reported as noncompliant. Ten of the 11 patients with IMD achieved full recovery, whereas 1 patient retained mild disability. No recurrent infections were reported.

## Discussion

This study represents the largest analysis to-date evaluating antimeningococcal protection strategies in patients undergoing C5 inhibition for renal indications. By combining longitudinal data from ERKReg with supplementary cross-sectional information from specialized European centers, we compiled a comprehensive and internationally representative cohort in a real-world setting.

The study identified several unique and surprising findings. Notably, despite current recommendations advocating for vaccination alone, nearly 60% of patients in European specialized centers were prescribed continued antibiotic prophylaxis in addition to vaccination. Combined protection was applied particularly in young children.

Booster coverage was notably low in our cohort, with MenB rates lower than MenACWY, yet even the latter not exceeding 40% of eligible patients. This gap likely reflects challenges in long-term adherence to vaccination guidelines, possibly because of unawareness of booster schedules years after initial vaccination. Moreover, the late introduction of vaccine recommendations,^7^ together with the lack of clinical studies, may further contribute to the observed variability in maintaining long-term immunity in this high-risk population.

Although vaccination was straightforward to monitor because it was performed by treating physicians, antibiotic prophylaxis depended on patient self-administration, making compliance more difficult to track. In our cohort, antibiotic noncompliance was reported in one-quarter of patients. This rate of adherence compares favorably with observations from the broader literature, where compliance to long-term antibiotic therapies is often as low as 40% to 60%, even among high-risk populations.^10^

An unexpected finding in our cohort was the association between advanced CKD stage at last follow-up and the use of single rather than combined protection. One might anticipate that patients with more severe kidney disease would receive broader protection. However, this pattern may reflect underlying demographic factors rather than clinical decisions. Adult-onset patients, who were less frequently administered combined protection and more prone to progression to advanced CKD, may have disproportionately contributed to the single protection group. Further studies are needed to determine whether this pattern reflects systematic practice differences in long-term preventive care.

The observed overall IMD incidence rate of 0.74 cases per 100 PY in our cohort aligns with previously reported rates, falling between the global postmarketing rate of 0.33 per 100 PY and the clinical trial estimate of 0.83 per 100 PY.^11^ Adjusting for antibiotic noncompliance brought a major impact on the findings in our study. Although the incidence rate of IMD did not differ among patients with prescribed combined and single protection, a 6-fold lower infection rate was observed in the combined protection group after adjusting for antibiotic compliance. The Kaplan–Meier analysis further supported this finding, showing a significantly higher infection-free survival in patients with combined compared with single protection (log-rank *P* = 0.047; Figure 2). In regression analyses using Cox models with Firth’s penalization, the observed difference in infection risk between protection types was confirmed in univariable analysis but did not remain statistically significant after adjustment for concomitant immunosuppression or primary diagnosis. Immunosuppression was recorded in a binary format and therefore could not capture variations in regimen intensity or duration, potentially diluting the effect of protection type. This limitation, together with the very small number of IMD events, likely contributed to wide CIs and reduced power in multivariable models. Comparable results were reported from the International Paroxysmal Nocturnal Hemoglobinuria Registry, where additional antibiotic prophylaxis did not measurably reduce meningococcal infection rates among vaccinated eculizumab-treated patients. However, interpretation was constrained by the small number of infections (*n* = 7) and missing data on antibiotic compliance.^12^ Larger cohorts with longer follow-up will be required to determine whether the observed trend represents an independent effect of protection type.

Among patient subgroups, kidney transplant recipients are of particular interest. Despite lifelong exposure to immunosuppression and C5 inhibition, only a single case of IMD was observed in this subgroup, occurring in a patient maintained on single protection. Although the small sample size limits definitive conclusions, this finding is consistent with a potential advantage of combined protection and emphasizes the need for clearer guidance in this high-risk population.

Among vaccinated patients with IMD, it is noteworthy that only 2 of 8 had received appropriate booster doses. Breakthrough infections tended to occur around 5 years after C5i initiation, pointing to a critical window in which waning immunity may predispose to infection if booster schedules are not strictly maintained. Moreover, infections in patients with implemented single protection tended to occur earlier, around 4 years after treatment initiation, whereas the only case in the combined protection group developed infection much later, after 11 years of C5 inhibition. Although limited by small numbers, this temporal pattern suggests that sustained combined protection may prolong the infection-free interval and mitigate late risk under long-term complement blockade.

The IMD cases in our study share similarities with those observed in otherwise healthy individuals in Europe, particularly with respect to age at infection and serogroup distribution, but differing in clinical presentation and outcomes. The median age at IMD onset in our cohort was 17.7 years, aligning with the incidence peak among otherwise healthy adolescents and young adults. Although no NG *N meningitidis* infections were observed in our cohort, this likely reflects regional epidemiology, because serogroup B remains the predominant cause of IMD across most of Europe.^13^ Previous reports indicate that NG strains, often associated with asymptomatic carriage, may account for some breakthrough infections in recipients of C5i.^3^ Because NG strains are not targeted by current vaccines, their occurrence provides a compelling rationale for maintaining antibiotic prophylaxis even in vaccinated patients and supports the concept of dual protection.

The clinical presentation of IMD in our study was broadly consistent with classical patterns, with fever being universal and headache, nausea, and vomiting being the most frequent. Other manifestations, such as neck stiffness, photophobia, altered mental status, and rash were less consistent, emphasizing the diagnostic challenge and the need for heightened vigilance in patients receiving C5i. Encouragingly, patients affected by IMD in our cohort exhibited favorable clinical outcomes, with 10 of 11 achieving complete recovery and only 1 exhibiting residual mild disability. This contrasts with the approximately 6% case fatality rate reported among C5i recipients in the United States^3^ and the 8% to 15% case fatality rate and substantial morbidity observed in the general European population.^13^ The predominance of mild clinical manifestations and favorable outcomes may reflect heightened awareness among families and health care providers, resulting in early diagnosis, and treatment initiation in this high-risk population.

In our cohort, antibiotic-resistant meningococcal strains were identified in 3 cases. All had been prescribed prophylactic antibiotics; however, only 1 was confirmed to be adherent. The emergence of resistant bacterial strains, such as penicillin-resistant *Streptococcus pneumoniae* in splenectomy patients, has been linked to prolonged antibiotic exposure. In addition, the risk of serious adverse events, such as macrolide-induced arrhythmias, complicates the rationale for long-term prophylaxis.^10^

The limitations of this study warrant consideration. First, its observational and retrospective design limits causal interpretation. Second, observer bias may have been introduced through physician-assessed compliance, particularly if knowledge of the infection influenced the assessment of compliance. This could lead to differential misclassification, possibly overestimating the association between noncompliance and infection risk. Although the small number of infection events reflects the rarity of these complications and is clinically reassuring, it limits the statistical power to detect associations with precision. Nonetheless, our findings indicate that in patients receiving long-term C5 inhibition, combined protection provides superior protection against IMD compared with single protection.

In conclusion, although current guidelines recommend single protection as the standard approach, many clinicians have adopted additional antibiotic prophylaxis to enhance protection in this high-risk population against potentially severe and life-threatening infections. Despite challenges such as suboptimal adherence to booster doses and notable levels of antibiotic noncompliance, our findings support that the combined approach of continuous antibiotic prophylaxis and vaccination offers superior defense against IMD in patients receiving long-term C5 inhibition. Continued prospective monitoring in clinical registries will be essential to define the optimal strategy of antimeningococcal protection in patients receiving complement inhibiting therapies.

## Appendix

### The ERKNet TMA Working Group (list available at erknet.org) and Other Collaborators listed below

Austria: Klaus Arbeiter, Fabian Eibensteiner (Vienna). Croatia: Lovro Lamot (Zagreb). France: Justine Bacchetta, Lisa Condamine, Dounia Habchi, Lydia Slimani, Amina Talhi, Sacha Flammier (Lyon); Marc Fila, Julie Tenenbaum (Montpellier); Laurence Heidet, Kahina Saidoun (Paris Necker); Nabila Moussaoui (Toulouse). Germany: Stefan Kohl (Cologne); Susanne Schaefer, Jose Antonio Ramirez Garcia, (Heidelberg); Marcus Weitz, Kathrin Buder, Lena Lechler (Tübingen). Great Britain: Matko Marlais (London). Hungary: Orsolya Horváth (Budapest). Italy: Giovanni Montini (Milan); Francesco Trepiccione, Miriam Zacchia (Naples-UOC); Germana Longo, Nicola Bertazza Partigiani (Padua); Antonio Gargiulo, Chiara Bettini (Rome, Ospedale Bambino Gesu); Vitor Hugo Martins (Turin, Regina Margherita Children’s Hospital). Poland: Aleksandra Zurowska, Magdalena Drozynska-Duklas (Gdansk); Anna Krakowska (Lodz). Portugal: Joaquim Calado, Rute Baeta Baptista (Lisbon); Ana Teixeira (Porto). Romania: Anca Marin, Valentin Mocanu (Bucharest). Slovenia: Andreja Aleš Rigler, Tomaž Šimnovec (Ljubljana). Spain: Carla Soto (Barcelona). The Netherlands: Romy Bouwmeester (Nijmegen); Eiske Dorresteijn (Rotterdam).

## Disclosure

FS received consulting fees from Samsung Bioepis for participation in Scientific Advisory Board meetings, with payment made to him, and from Alexion for consulting on pediatric trial programs in potential new indications for C5 inhibitors while participating in the Alexion Global aHUS Registry Steering Committee, with payments made to his institution. ALSL received consulting fees and speaker honoraria from Alexion and participated in the Alexion Global aHUS Registry; all payments were made to her institution. MP received speaker honoraria from Inozyme Pharma and Angelini Pharma. OB received speaker honoraria from Alexion and Samsung. MCM received speaker honoraria and a travel grant from Alexion. SH received a grant from the German Research Foundation (Collaborative Research Unit 329) awarded to her institution; consulting fees from Takeda, speaker fees from Alexion and the German Society for Pediatric Nephrology, and advisory board fees from Samsung, with payments made to her. All the other authors declared no competing interests.

## Supplementary Material

Supplementary File (PDF) STROBE Statement checklist.
